# Effect of a Virtual Environment on the Development of Mathematical Skills in Children with Dyscalculia

**DOI:** 10.1371/journal.pone.0103354

**Published:** 2014-07-28

**Authors:** Marcus Vasconcelos de Castro, Márcia Aparecida Silva Bissaco, Bruno Marques Panccioni, Silvia Cristina Martini Rodrigues, Andreia Miranda Domingues

**Affiliations:** 1 Nove de Julho University (UNINOVE), São Paulo, São Paulo, Brazil; 2 Technological Research Center, University of Mogi das Cruzes (UMC), Mogi das Cruzes, São Paulo, Brazil; 3 Technology College of Mogi das Cruzes (FATEC), Mogi das Cruzes, São Paulo, Brazil; University of Westminster, United Kingdom

## Abstract

In this study, we show the effectiveness of a virtual environment comprising 18 computer games that cover mathematics topics in a playful setting and that can be executed on the Internet with the possibility of player interaction through chat. An arithmetic pre-test contained in the Scholastic Performance Test was administered to 300 children between 7 and 10 years old, including 162 males and 138 females, in the second grade of primary school. Twenty-six children whose scores showed a low level of mathematical knowledge were chosen and randomly divided into the control (CG) and experimental (EG) groups. The EG participated to the virtual environment and the CG participated in reinforcement using traditional teaching methods. Both groups took a post-test in which the Scholastic Performance Test (SPT) was given again. A statistical analysis of the results using the Student's t-test showed a significant learning improvement for the EG and no improvement for the CG (*p*≤0.05). The virtual environment allows the students to integrate thought, feeling and action, thus motivating the children to learn and contributing to their intellectual development.

## Introduction

Mathematical knowledge is one of the foundations of human cognitive and creative development [1]. It is a basic tool for performing daily activities and encompasses various areas of knowledge. During childhood, mathematics is used for such tasks as dividing candy among friends, counting allowance money and tracking time while playing video games. During adulthood, mathematics is used for such tasks as tracking medication times and managing receipts, payments and finances [2], [3].

However, individuals commonly have some type of difficulty in acquiring mathematical skills. The Programme for International Student Assessment (PISA) classifies mathematical proficiency in six levels, with level 6 being the highest. Approximately 90% of the countries evaluated by PISA had 10% or more of their students at level 1 [4]. At this level the students are able to answer questions clearly defined involving familiar contexts and other relevant information. In sum, the students need to be able to identify information in explicit situations and also performing actions according to direct instructions [4].

Learning disabilities in mathematics include deficits in multiple mathematical abilities depending on the student's age and education level: counting in your head, number patterns, operations (addition, subtraction, division and multiplication), area and shape and size and measurement [5], [6].

Children with mathematical learning disabilities are classified as having Dyscalculia [7] if they present a specific deficiency in the acquisition of mathematical skills [8]–[10]. As consequence, different subtypes of numerical processing disorders can occur [11] and might be symptom of several disorders, including:

- Disorders in general cognitive processes, such as working memory, verbal ability and spatial vision [7], [12], [13], [14].Errors in the development of neural systems dedicated to number transformation [11], [15], [16].
- Deficits in quantity representation when reading numerical symbols [17].

Disorders broad enough to affect a generalised system dedicated to working with continuous and discrete sizes [18]. Children with mathematical learning disorders, especially children with Dyscalculia, may show a lack of motivation, feelings of guilt or math phobia [15]. Some children with mathematics learning disabilities are discriminated against or bullied by classmates, which further discourages them in a school environment [19]. They may drop out of school, thus affecting their ability to function in society. They may also avoid professional activities that use math in their adult life [20], thus affecting their professional and personal development [21]. However, these children are not “stupid or lazy” and are inside the normal intelligence pattern, but their brains process information in a different way [22]. Education experts believe that pedagogical strategies based on games may remediate the learning disabilities of these children, because the natural way for children to learn about the world around them is often through play. Experts believe that the use of computer games based on situational problems with pedagogical goals may stimulate the children's thoughts and generate positive attitudes, thus contributing to their intellectual development and motivating them to create their own strategies [23].

Children can teach themselves mathematical concepts and specific skills by playing [24]. Thus, if applied properly, computer games can encourage child involvement in the learning process. Firstly, because the games contain challenges that can motivate children to search for a solution to the presented mathematical problem [25], [26]. Secondly to stimulate the construction of logical-mathematical thinking processes [24], [27] while having fun. Therefore, the more stimulating the challenges, the more the children are interested in the game [11]. In addition, computer games facilitate self-correction by allowing the child to immediately see the result of his or her actions, which helps the child understand his or her errors. Thus, games can be used as a learning tool to promote child participation [27].

Because computer games are an object of curiosity, exploration and interaction for children, they can become an efficient pedagogical tool if developed with a pedagogical purpose and with well-planned activities. As result, the computer games can motivate, entertain and also facilitate the learning process by training mental functions and promoting the development of logical reasoning and thought [23], [28].

Many virtual environments and computer games have been developed with educational goals to help children with learning disabilities. Previous studies have shown positive results for children with learning disabilities in reading [29]–[33]. These studies showed that student performance has improved significantly by using computer-based games when compared with traditional computer-assisted teaching techniques [34].

Both entertainment and educational computer games addressing certain mathematical skills were found in the literature [25], [27], [35]–[38], but these games did not cover mathematical thinking in its entirety. Only two of these games were used to remediate Dyscalculia: the game “Number Race,” which was developed to improve basic numerical cognition [38], and the game “Graphogame-Math,” which was developed to improve exact numerosity and number symbols [39]. An adaptive E-learning tool that teaches with an entertaining numerical comparison task [40] was also tested on children with mathematical learning disabilities. The results obtained from these computer-based methods show that they may be useful for remediation of Dyscalculia in children.

However, there are not many studies on interventions that both remediate Dyscalculia and promote participant discussion on a specific problem, combining the games' entertainment resources with an educational environment [41].

This article presents a virtual environment that incorporates 18 educational computer games, which are designed to contain elements of entertainment games, into a fun theme [41]. This virtual environment addresses the mathematical skills necessary for an individual to function in society. We also show the effectiveness of this virtual environment as a tool to minimise the persistent difficulty in learning math that occurs in children with Dyscalculia.

## Materials and Methods

### Description of the virtual environment

The virtual environment used in this study, named “Tom's Rescue”, was developed with the intention of generally stimulating reasoning in children by generating positive attitudes. In addition, it was also developed to confront the children with fun situational problems, previously planned with the pedagogical goal of helping, to children develop necessary mathematical skills for functioning socially.

This virtual environment is composed of two settings, a city and a forest, which contain objects that are linked to entertaining and educational mini-games. The storyline of the virtual environment was designed to promote child interaction and follows a character named Tom. Tom is a small turtle from a community located in a forest near the city who was captured to be sold in an open-air market in the city. Some members of his community are upset at Tom's capture and plan a rescue, which is led by a monkey named Caco. Caco is the avatar controlled by the child using the keyboard.

**Figure 1:**
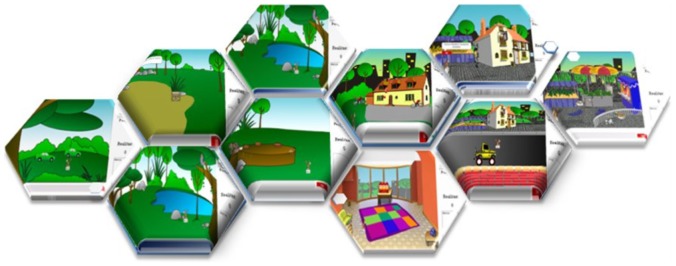
Diagram of the - Virtual Environment's scenes.

The environment is accessed with an Internet Explorer-compatible web browser. On the first visit, the child must enter a user name. This name will identify them in the virtual environment and will be the name of their main character. The child then explores the environment to find the various links to the mini-games (Figure 1).

**Figure 2:**
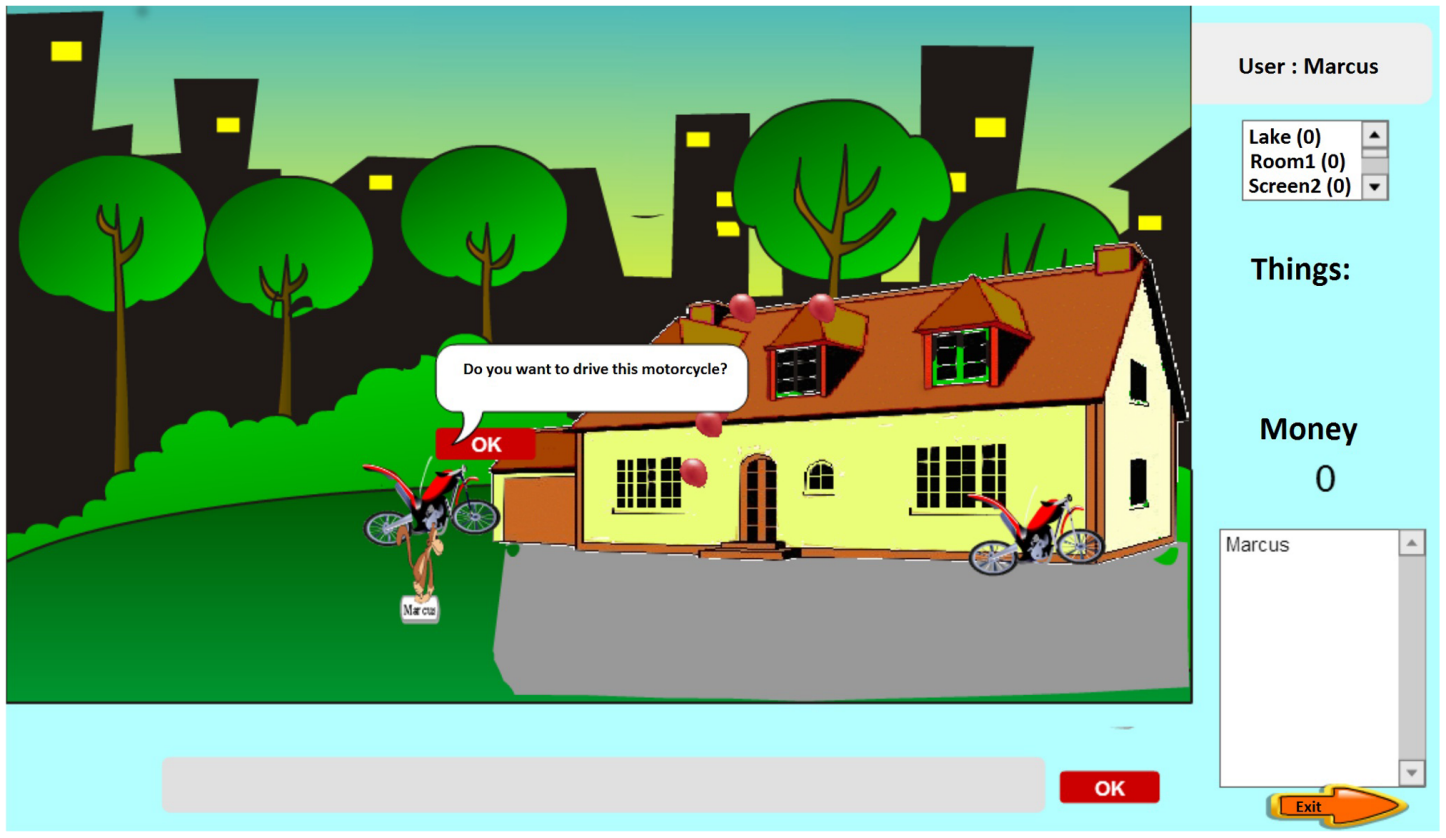
Screenshot of one of the scenes in the virtual environment.

When the avatar is positioned near an object (a tent, door, car, etc.) that links to a game, an invitation message appears (Figure 2). The child can ignore the invitation by moving the avatar to another area or can easily accept the invitation and be transported to a mini-game focused on one or more mathematical skills. The games accessed through the links were developed by the author with consideration of the intrinsic motivation factors that should be in computer games as challenge, control, feedback, curiosity and fantasy [42]. We was also assumed that Dyscalculia is a deficit in the cognitive representation of numerosities [11], [43]. To remediate learning disabilities in mathematics for children with Dyscalculia, who have a persistent difficulty processing numbers, we considered, cases of developmental Dyscalculia with no evidence of cerebral trauma that could not be explained entirely by deficiency or inadequate education [44], [45]. It was also taken into account to work with visual and spatial memory can improve students' ability in mathematics [46].

According to the literature, children with Dyscalculia may be incapable of visualising groups of objects within a broader group, making comparisons between smaller and larger objects, saying a sequence of numbers in increasing or decreasing order, understanding the signs of basic operations and resolving a relatively simple equation [7]. They may also have difficulty understanding the significance of numerical representations and comparing numbers and must rely on immature strategies (such as counting one by one and/or counting with their fingers) to complete arithmetic procedures. They may also have difficulty coming to conclusions based on relatively simple concepts, rules, formulas or mathematical procedures [8].

Therefore, to make up the virtual environment, games that cover number identification, numerical sequences, number comparisons, the concept of even and odd numbers and the four arithmetic operations (addition, subtraction, multiplication and division) were developed. Table 1 and Table 2 show the cognitive and mathematical skills covered by the games that make up the virtual environment. Table 3 presents a description of these games.

**Table 1:** List of cognitive skills addressed by the games developed by the author.

Cognitive skill	Games developed by the author	Figure
Working memory	Memory and Flash Cards	Figure 3
Spatial visualisation	Caco Flies, Off Road, Shark, Beware of the Alligator, Hit the Balloons, Motocross and Number Race (1 and 2)	Figure 4
Quantity representation, using number symbols	Lost Ruins, Wrapping Gifts, Harvesting Apples and Hit the Balloons.	Figure 5
Processing continuous and discrete quantities	Caco Flies, Lost Ruins, Hit the Balloons, Off Road, Number Race 2, Dance, Dance and Dance!!, Shark, Selling Corn, Beware of the Alligator, Motocross and Wrapping Presents.	Figure 6
Reading and writing numbers	Lost Ruins, Off Road, Shark, Selling Corn, Wrapping Presents and Hit the Balloons	Figure 7
Constructing the significance of the number from its various uses in society	All of the games	All Figures
Calculation procedure development	Caco Flies, Lost Ruins, Slot Machine, Off Road, Number Races 1 and 2, Shark, Selling Corn, Beware of the Alligator, Flash Cards and Dance, Dance and Dance!!	Figure 8
Recognition of measurable quantities	Lost Ruins, Slot Machine, Off Road, Wrapping Presents, Shark, Hit the Balloons, Selling Corn, and Beware of the Alligator	Figure 9

**Table 2:** List of games developed by the author and maths skill(s) addressed.

Game	Maths Skill(s) Addressed
Caco Flies	Arithmetic operations (addition and subtraction), visual reasoning, mental calculations.
Lost Ruins	Arithmetic operations (addition and subtraction), visual reasoning, counting, numerical size, mental calculations.
Slot Machine	Addition, comparing geometric shapes.
Off Road	Number sequences, subtraction and addition, mental calculations.
Number Race 1	Counting and number sequences.
Number Race 2	Arithmetic operations (Addition), mental calculations.
Memory	Visual reasoning, memory.
Shark	Addition, mental calculations.
Selling Corn	Addition, multiplication, mental calculations.
Beware of the Alligator	Addition, mental calculations.
Flash Cards	Addition, mental calculations.
Noggin Breaker	Visual reasoning, visual space.
Monkey Puzzle	Visual reasoning, visual space.
Motocross	Symbol identification, even and odd numbers.
Wrapping presents	Counting, geometric shapes.
Dance, Dance and Dance!!	Arithmetic expression (addition), mental calculations.
Apple Harvest	Symbol identification, visual reasoning.
Hit the Balloons	Visual reasoning, visual space, counting.

**Table 3:** List of games developed by the author and its description.

Game	Description
Caco Flies	While the avatar (‘Monkey Caco’) is piloting the airplane, the avatar should collect the birds on the sky or in the grass labeled with numbers which is the results from the mathematical expression presented on the top of the screen. If the avatar does it correctly while the time is running out, the score will be automatically increased while travelling. Otherwise, the score will be systematically reduced and the airplane will lose control until ending with game over.
Lost Ruins	This game consists of a maze that presents obstacles and puzzles subdivided into 3 different phases. In the first phase, the user should see on the screen the number displayed and then count sequentially the pieces of the ladder until identify the exactly position order which matches with the number previously displayed. Once the user “guesses” it correctly, the avatar will be able to climb to the above floor. In this stage, known as second phase, the avatar (‘Caco’) should collect the boxes with eggs. In this moment, the number of eggs inside the box will be displayed and the user should again to identify the right position from the number displayed by the game on the screen as previously described. Once the user has completed this phase, the game will be automatically advanced to the next phase (phase 3). In the third and last phase, the monkey Caco needs to pick the bananas, but for that the user should answer correctly the mathematical questions presented on the screen every time that Caco finds a bunch of bananas.
Slot Machine	Slot Machine - The child can invest the money earned in other games located in the game room of the City scenario.
Off Road	Off Road – Racing game where the child should control a truck to get to the end of the trail overcoming obstacles. If one reaches some pineapple, shall inform the result of a mathematical expression and if hit earns points and can continue playing. If one does it wrong nothing happens and one can keep trying until get it right. To start the game, the child must turn off the alarm system, hitting a number between one and ten randomly selected by computer. Every child attempt, the system provides a hint written and spoken (generated by the speech synthesizer), indicating if the secret number is higher or lower than that reported. In this game, the child can hit through trial and error, but it is expected the child realizes that it is important to associate the response of each result to move faster on the track. The alarm sounds if the child exceeds three attempts, but the child can try again at another time.
Number Race 1	Racing game where the child should control a car and pass upon the right sequence of numbers. Otherwise, the car skids and lives are lost.
Number Race 2	In this game, the child must pass only upon the right arithmetic accounts not to skid.
Memory	The child must use logical reasoning and memory to find the position of a card with the same number of chosen one. By clicking on a card, besides seeing the number, the child also hears its sound and, from this moment, the child must click a second card. If the number on it is equal to the previous one, the two disappear. Otherwise, nothing happens and the child should continue looking for another card with the same sound that initially clicked.
Shark	In this game the ability to add is crafted in getting the result always equal to five. The child must control the shark (in the avatar Caco is ridden) on the seabed for it to eat the right fish, avoiding the drowning of Caco. Each fish represents a number. During the game, some fish appear with hidden numbers, this happens to arouse more emotion, as if the child chooses a fish without number, the lucky will be at risk.
Selling Corn	Selling Corn – The child must act on Caco avatar for him to sell corn in a tent. Customers ask what the total amount they have to pay leading a number of corns drawn by the computer. In this game it is intended that the child, through mental calculation, is able to respond to customers which the total amount payable in relation to the requested amount of corn. The more play selling corns in tent, the more earn money.
Beware of the Alligator	During the exploitation of Forest scenario, the child will find a lake where the Avatar (Caco Monkey) will guide its friend Alligator eating fish. At this time, the alligator is directly controlled by the child. This game is intended to work with the sum with the result 5. Alligator is expecting the right fish pass at an appropriate time, it should eat them so that the sum is equal to 5.
Flash Cards	In this game the child chooses a card with a mathematical expression and associates it with another card that displays the result, eliminating them and earning points. To every game session numbers involved in the cards are randomly generated. The child has a time of 60 seconds to solve the problems, which decrease according to the evolution of each phase. If the child cannot hit the cards in the estimated time, the game offers a new session with other numbers drawn.
Noggin Breaker e Monkey Puzzle	These games focus on memory, reasoning and observation to assemble puzzles with images of low complexity.
Motocross	In the first phase, the child should ride a bike and grab stars with the number requested by the ant (on the top). If the child picks the wrong number, the fox advises that it is not the correct number, otherwise earn points. In the second phase, even and odd numbers are worked and the child avoids the odd stars and rescues the even stars, earning points. During training, the Avatar Caco gets the ant's help, which informs what number should be taken. If the child takes a star with wrong number, lives are lost.
Wrapping presents	The Caco Avatar at first person should prepare decorative boxes according to customer's request, which gets upset with the care delay. The more clients are met, the more money is earned. We are working on this game counting, shapes and geometric figures, letting the creative flowing for decorating a box the way one finds most interesting, since it meets the customer's request. In this game, the clients do not repeat as there are a combination of hats, clothes, mouths, noses and eyes, creating a large number of different clients.
Dance, Dance and Dance!!	The Avatar must dance in various styles according to the number of correct answers. For this, one must collect the trays with the right mathematical expressions and destroy the trays with the wrong mathematical expressions using a weapon that emits laser. If collecting the wrong tray or destroying a right tray, one will lose points and thus Caco will dance less, stopping its movement if it has a number of points equal to zero.
Apple Harvest	The Avatar needs to collect apples with the number requested by the fox (in writing and spoken), avoiding apples labeled with other numbers that make the score decrease. The apples should be collected in a shortest possible time to earn more points. Purposely, the numbers are presented in the game in increasing order to also work the counting.
Hit the Balloons	The child must pop the balloons by hitting them with a small ball with nails and in the end the remaining balloons are counted to change phase. The game consists of 10 levels and allows the child to repeat the activity several times, until getting the right amount, without emphasizing the error.

**Figure 3:**
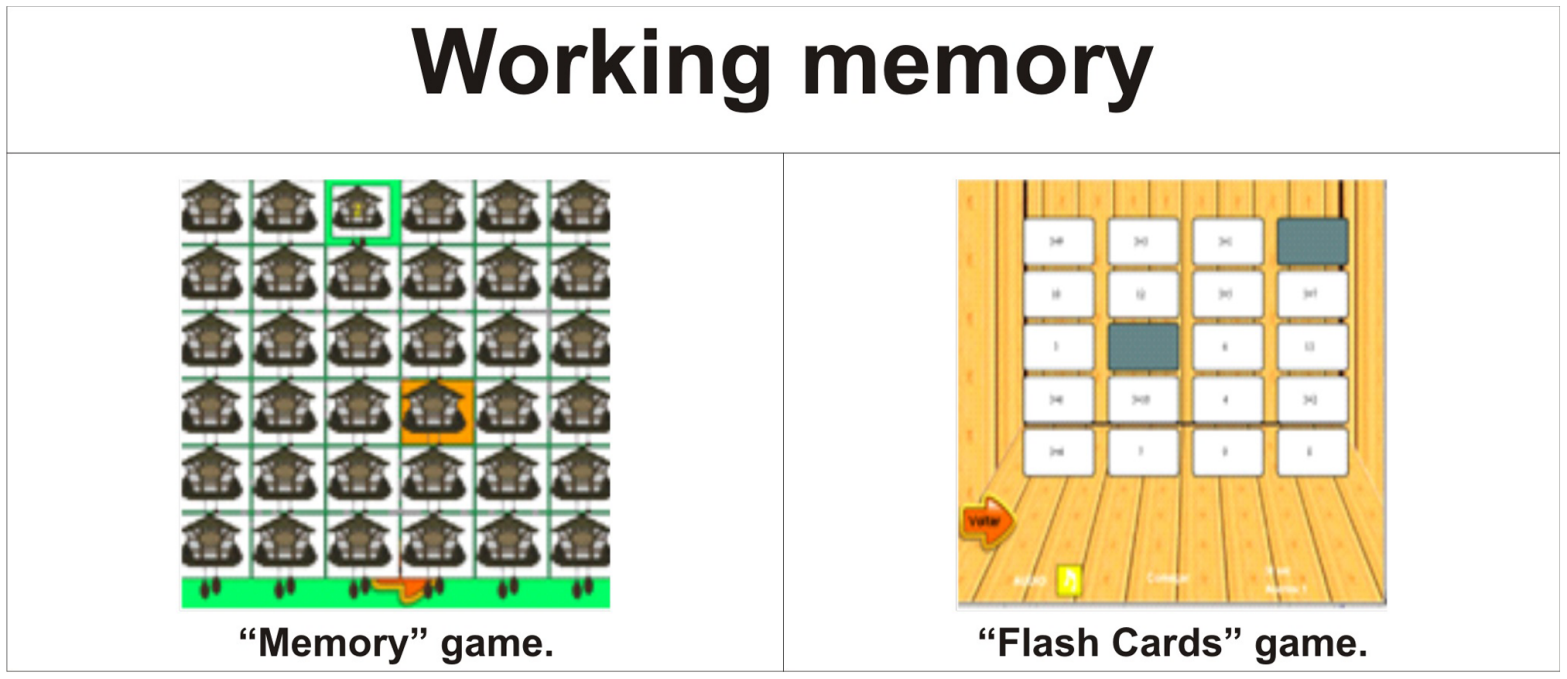
Working memory.

**Figure 4:**
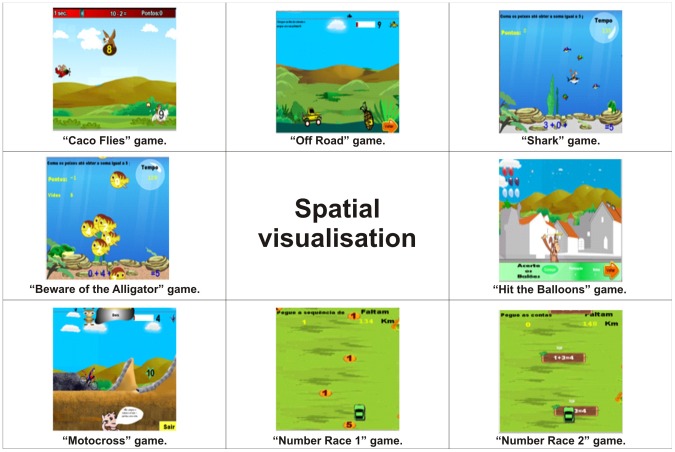
Spatial visualisation.

**Figure 5:**
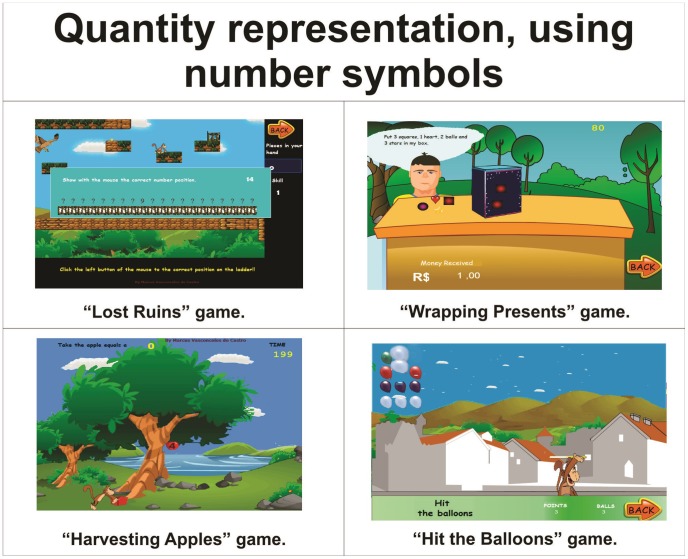
Quantity representation, using number symbols.

**Figure 6:**
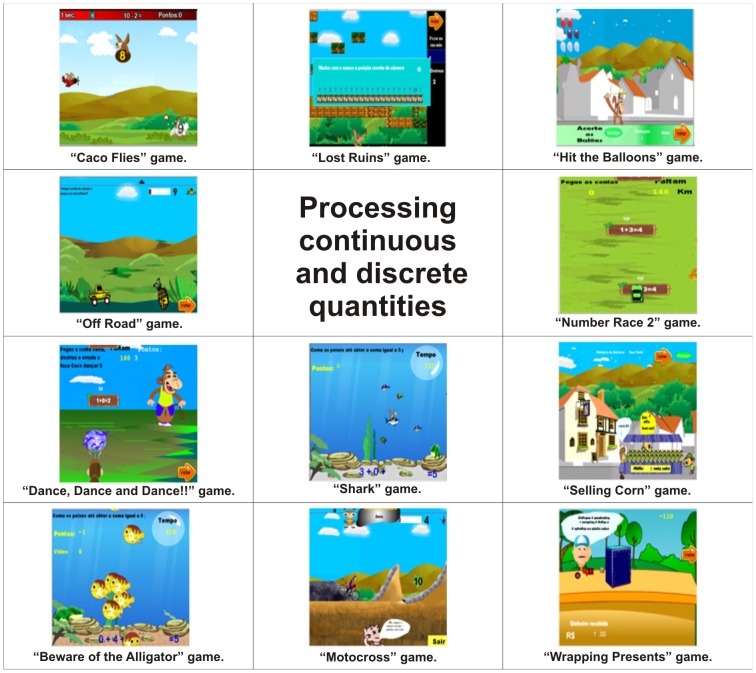
Processing continuous and discrete quantities.

**Figure 7:**
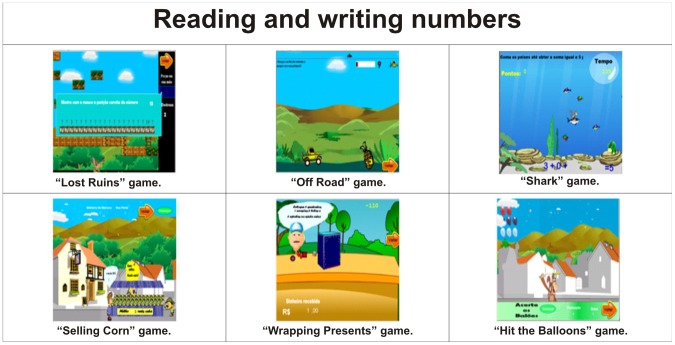
Reading and writing numbers.

**Figure 8:**
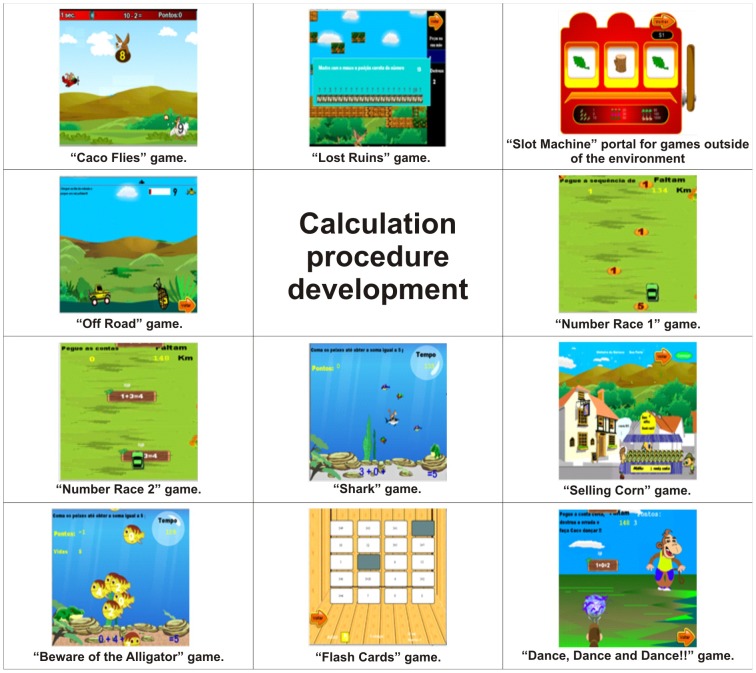
Calculation procedure development.

**Figure 9:**
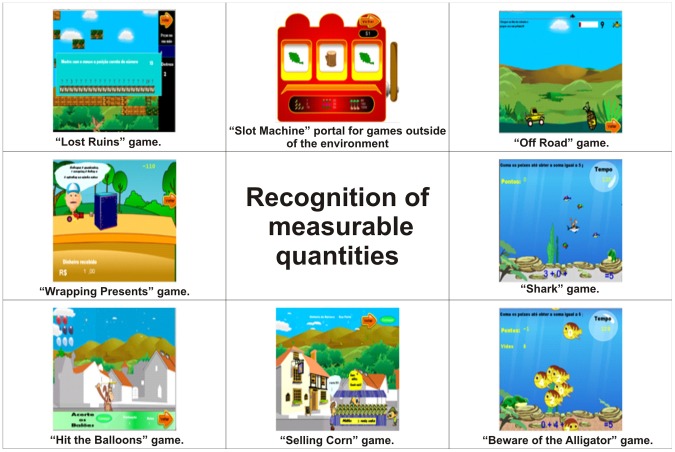
Recognition of measurable quantities.

As a structure of cognitive skills for the profile of the games there are Working memory in Figure 3, Spatial visualisation in Figure 4, Quantity representation, using number symbols in Figure 5, Processing continuous and discrete quantities in Figure 6, Reading and writing numbers in Figure 7, Constructing the significance of the number from its various uses in society in all Figures, Calculation procedure development in Figure 8 and Recognition of measurable quantities in Figure 9.

The virtual environment was implemented in a way that does not force the child to play the games in a linear order. They can try the most difficult game, then go to the easiest and return to any game that they want by moving their avatar around the virtual environment.

In summary, each of the games developed or adapted by the author to make up the virtual environment first seeks to link entertainment with educational goals, so that the child is initially attracted by the entertainment. Then, the challenges implemented in each game can stimulate the child's desire to win and, consequently, stimulate his or her cognitive development and interest in mastering specific mathematical skills through different strategies.

### Ethical principles

Before to start this research with the specific, this research project was approved by the Ethics Committee in Research involving humans at the University of Mogi das Cruzes (CAAE-0073.0.237.000-010, process CEP/UMC-078/2010).

All the participants were adequately informed about the aims, anticipated benefits, and methodology of the study, as well as about the privacy of research subjects and confidentiality of their personal information, and institutional affiliations of the researcher. As the research procedures are non-invasive, does not cause pain or discomfort to the children enrolled in the study, written informed consent were signed by the experts, teachers, director of the schools involved, and also by the guardians on behalf of the children.

### Participants

Three hundred children participated in the study, including 162 males and 138 females. The children were between the ages of 7 and 10 years, with an average age of 8 years and a standard deviation of 0.63 years. All children were in the second grade of public primary school in the eastern region of the city of São Paulo, Brazil.

In addition, according to the information supplied by school and their teachers, all children had: the same socioeconomic level; their homes near the school; the same learning opportunities; computer literacy; already used computerized game in the computer classes of school; and normal intelligence.

A 60-minute pre-test was given at the beginning of the study. For this test, we used the arithmetic test contained in the Scholastic Performance Test (SPT), which is a standardised test administered in Brazilian schools to evaluate a child's ability to solve mathematical problems verbally and perform written arithmetic operations [47]. The children were classified as “high performing” for SPT scores greater than 13, “average performing” for scores between 10 and 13 and “low performing” for scores less than 10.

**Figure 10:**
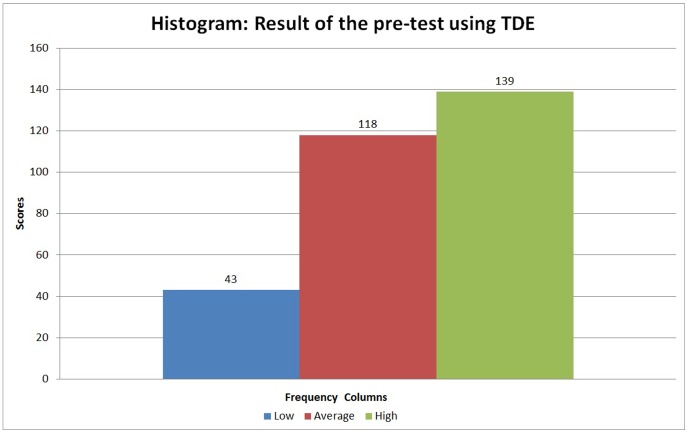
Histogram of performance determined by scores on the SPT pre-test administered to 300 children.

The results of the pre-test led us to select 43 children who scored less than 10 on the arithmetic test, as illustrated in Figure 10. Of the 43 children with low SPT scores, only those who could be classified as having Dyscalculia based on the literature were selected. According to the literature, between 4% and 8% of school age children have dyscalculia [48]–[51] and have chronic difficulties learning math [21]. Therefore, we selected 26 (8.7%) children whose teachers confirmed that they had persistent difficulties learning mathematical skills, even with reinforcement given during regular classes [21] in the first and second grade in the school.

Children were excluded if they often missing classes at school or could not perform the procedures of the intervention or reinforcement outside the normal class time, and also had behavioural problems or had psychiatric, neurological or other pathologies that were identified during the interview or previously noted by their school [52], such as learning disabilities in reading and writing, Dyslexia, Dysgraphia, ADHD, etc.

The children's school performance supplied by their teachers and school are based on SARESP (The Assessment System of School Achievement in São Paulo State), an achievement tests administered by the public school during the school year to meet standards set by educational policies. The SARESP's results allow to teachers identify children with similar performance in reading and writing and persistent learning disability in math [53], [54].

Additionally, the scores on intelligence tests (IQ) were not used in this research because according to the literature: Developmental Dyscalculia (DD) is characterized as a specific disorder that affects the normal acquisition of arithmetic skills in children with normal intelligence and adequate educational opportunities [21], [48], [55]–[57]; the learning difficulties associated with persistent low achievement in mathematics is not attributable to intelligence [51]; the scores on IQ tests is not considered “as risk factors for Dyscalculia” [58]; and also the “scores on IQ tests are irrelevant and not useful and may even be discriminatory” [59].

According to their responses on the SPT, the 26 selected children were unable to say a number sequence in increasing order [7], [43], had difficulty understanding the meaning of numerical representations or mathematical symbols [7], [43], had difficulty processing numbers [7], [43], had difficulty translating between verbally stated numbers and Arabic numerals [55], [60] and made mistakes choosing calculation procedures, which could indicate a problem with processing symbols or in the execution of arithmetic calculation procedures [61]. The selected children were also unable to solve simple arithmetic exercises [8], [21]. The children were then randomly divided into two groups, experimental (EG) and control (CG), which were matched for age (average of the EG = 8.08 years and CG = 8.15 years), sex (both groups had 8 boys and 5 girls) and SPT score (EG = 2.85 and CG = 2.62).

### Intervention phase using the virtual environment

Both groups (EG and CG) had interventions in addition to their regular classes; the EG interacted with the virtual environment, and the CG participated in a reinforcement using traditional teaching techniques performed by the same teacher from their regular classroom. The students of CG group solve math problems covering the same mathematical skills implemented in the virtual environment used by the GE group.

During the intervention stage with the virtual environment, 100% of the children of EG group were taken to a room with networked computers at the school where they usually had computer classes, and they were aware that they were going to play on the computers. The virtual environment sessions lasted approximately 60 minutes and were given twice a week for 5 weeks [52]. The children's interactions while playing were observed and recorded.

On the first session of the intervention, the researcher presented the virtual environment to the children, explaining about the challenge in each game and informing them that they could use the mouse and also the directional arrow keys to explore the virtual environment and to act on the avatar. He also explained that the time spent in each game would be not computed, the interaction would be self-explanatory and when an error occurred, the children could start the game again. Afterward was no longer any interaction between the researcher and the children.

All of the children actively participated in each session, exploring the virtual environment and freely choosing the games that they wanted to play. When one child found a game, he or she used chat to tell the other show to find it, and the other children also played that game. In the first session, the children explored the environment, played the games Harvesting Apples, Number Race, Add Up and Wrapping Presents and used the chat function. In the second session, they found the game Hit the Balloons and played Number Race again. In the third session, the games Sharks and Motocross were quickly found and played by all. In the fourth session, the game Flash Cards was found, and the children competed to see who could solve the cards first.

In the fifth session, the game Slot Machine was played by some of the children, and one child played Wrapping Presents. Other games (already discovered) were also played again. In the sixth session, the children played the games Lost Ruins, Selling Corn, Noggin Breaker and Beware of the Alligator in rapid succession. The children also played the games Motocross, Flash Cards and Wrapping Presents.

In the seventh session, the children found the game Dance, Dance and Dance!! and played it for several minutes, but most of the children kept playing Motocross and Off Road, while one child preferred Wrapping Presents. In the eighth, ninth and tenth sessions, the children initially played the games Motocross and Off Road, then played practically all of the games over the course of these sessions and continued to explore the environment in search of new games.

### Post-test

After the intervention with the virtual environment, the arithmetic test contained in the SPT was administered again to the EG and CG groups as a post-test. Afterward, to guarantee the digital inclusion of all children, access to the virtual environment was opened up to all children in the CG and the other children that had taken the pre-test.

### Statistical Analysis

To verify that the data are parametric or nonparametric was used the D'Agostino test (for N≥10 sample k). Additionally, the control of independent variables was performed through the inclusion criteria that already contemplate no deviation of the variables. The results from the pre-test and post-test were analysed to compare the data from the EG and CG group. To check for significant differences between groups, confirming or not if the virtual environment had enhanced the math proficiency level in the children of GE group it was used a Student's t-test, and the results were considered significant at or below 5% (*p*≤0.05).

## Results and Discussion

**Figure 11:**
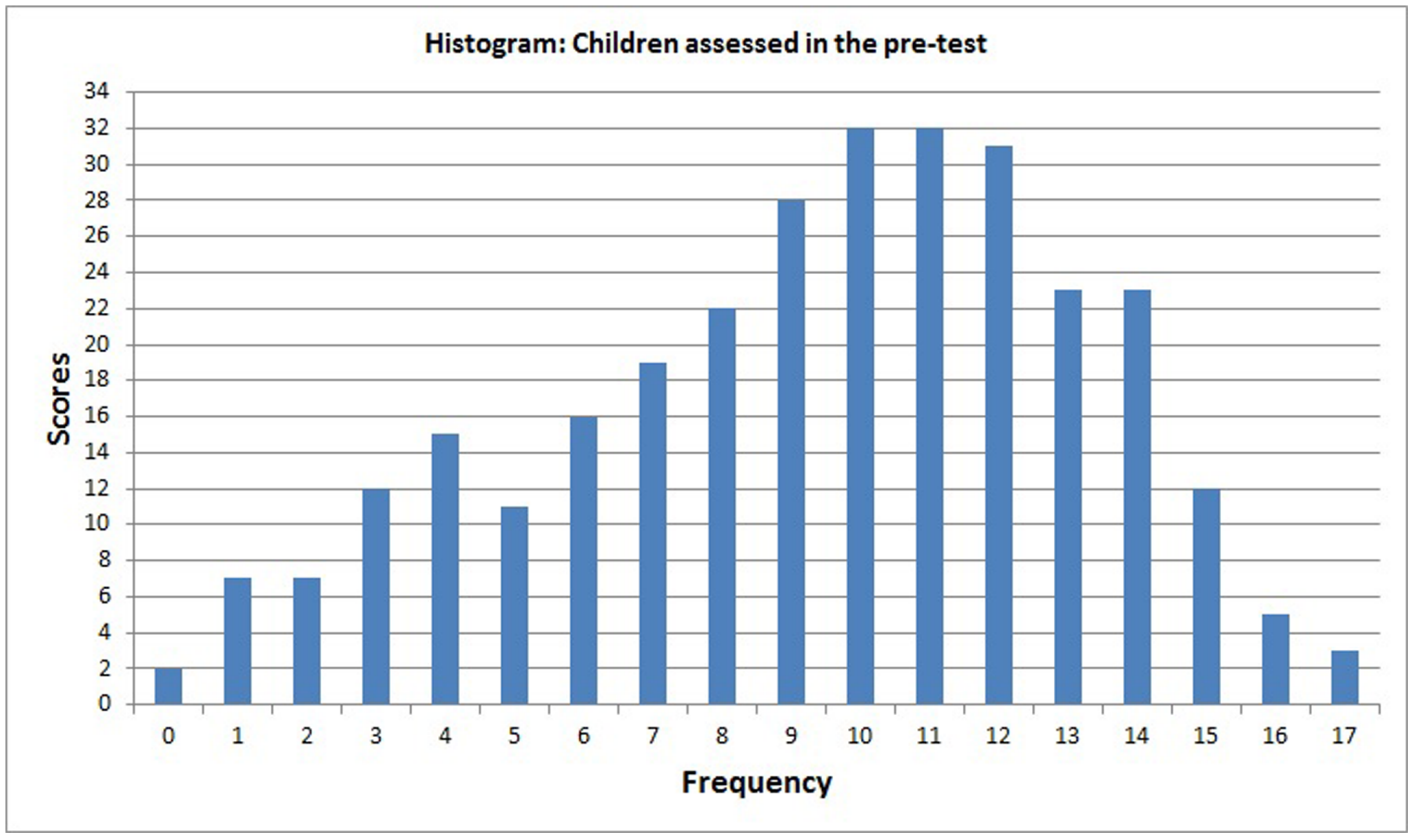
Histogram showing the pre-test scores for the 300 tested children.

**Figure 12:**
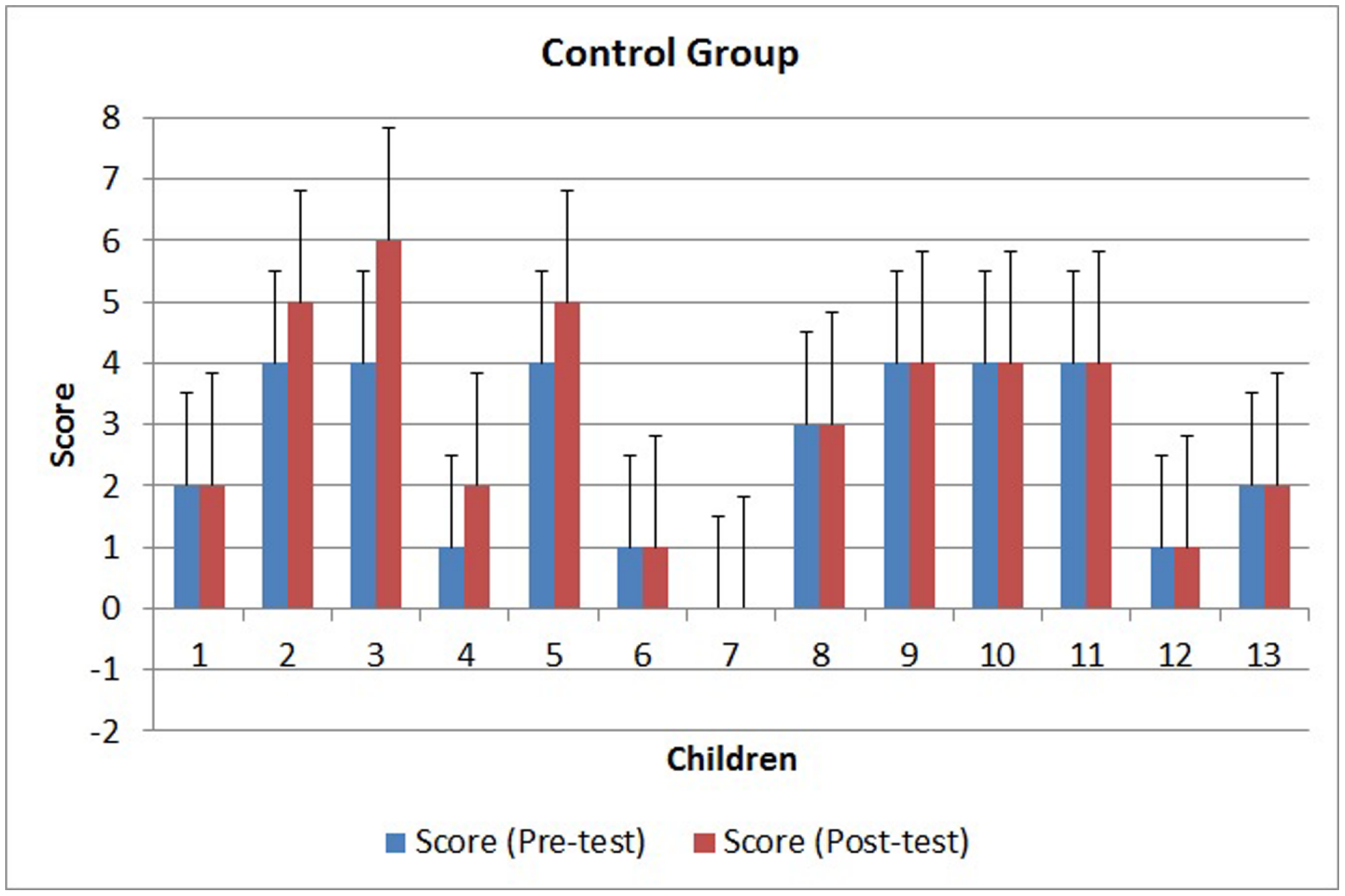
Scores for the control group on the pre-test and post-test.

**Figure 13:**
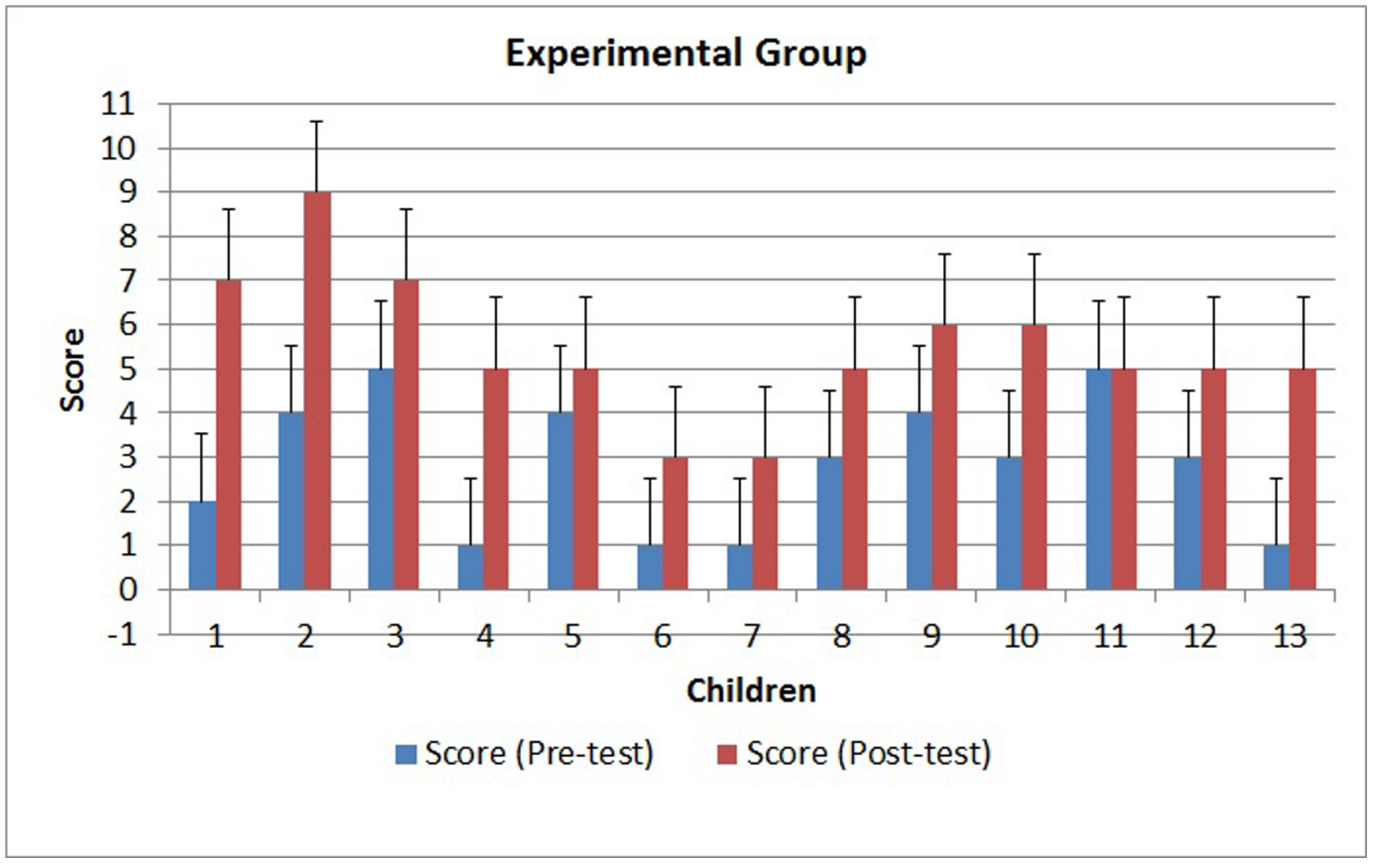
Scores for the experimental group on the pre-test and the post-test.

**Figure 14:**
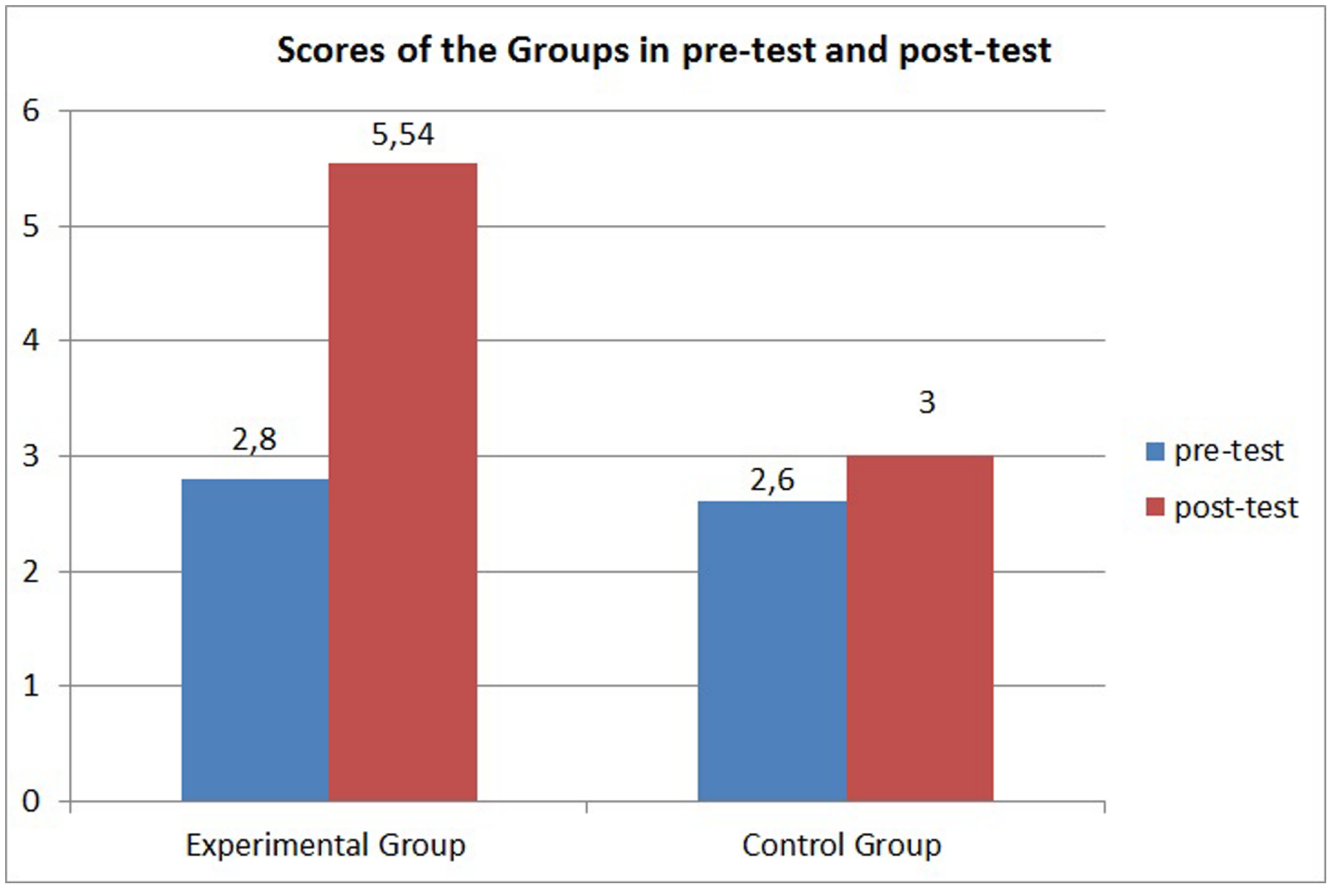
Average scores for the experimental and control group on the pre-test and the post-test.

The results obtained from the tests and statistical analyses are shown in Figures 11 to 14. Figure 11 shows the scores obtained by the 300 children on the pre-test. The results of the post-test for the CG and EG groups are shown in Figures 12 and 13. Figure 14 shows the average score for each group on the post-test and pre-test.

Analysis of the scores obtained by the 300 children on the arithmetic subtest (Figure 11) shows an average score of 9.35, with a standard deviation of 3.8 and a variance of 14.7. The pre-test results show that some children performed excellently for their grade level (a score above 13) while others performed badly (a score below 9).

According to their teachers, 61% of the children who performed badly on the arithmetic subtest had persistent difficulties learning math during their traditional classes. These children were chosen to test the virtual environment, which was developed to help children with this type of learning disability.

Statistical analysis using a Student's t-test on the pre-test scores obtained by these children (randomly grouped as CG and EG) showed that the difference between the groups was not significant before the intervention (p = 0.7533). In other words, the two groups had similar proficiency levels in mathematics before the intervention.

However, statistical analysis of the scores of the two groups (EG and CG) on the post-test showed a significantly different proficiency level between the groups. The intervention using the virtual environment yielded a significant score improvement for the EG (p<0.0001), whereas the CG did not show a score improvement (p = 0.0543). As shown in Figure 14, the average score on the post-test for the EG (5.09) was higher than the average score for the CG (3), indicating a positive effect of the virtual environment.

The improvement in scores for the EG probably occurred because the children tried to perform analyses and predictions by acquiring mathematical skills and using mathematical thought processes to resolve a specific problem or challenge that they encountered. This process may promote the development of abstraction, a cognitive skill essential for mathematical understanding [24].

Additionally, the children could freely explore the virtual environment and interact with the games without a pre-established sequence, playing the same game several times to beat it. The virtual environment did not have a mechanism to punish the children for incorrect answers. Thus, the frustration of losing was avoided, and the children could replay the games as they gained mathematical skills and their determination to beat the game grew. A design that avoids frustration and allows free exploration is very important for children with a history of frustration in the classroom.

According to reports from parents and teachers, the children were excited to participate in the training. They were also happier, showed more effort in solving math problems in the classroom and were more autonomous and willing to continue their studies. For example, one of the children that had persistent difficulties learning mathematics brought the workbook that she had performed in class and proudly showed a note from the teacher praising her correct answers.

The act of playing appears to have enriched the teaching-learning process of this child, helping her to develop a mathematical way of thinking as observed in literature [23], [24], [27], [28]. The act of playing permitted this child, as well as the others, to reflect, make predictions and compare objects, events and attitudes. These aspects of playing increased the children's experience and promoted the development of skills and competencies useful in daily life, as observed by [23], [24].

The freedom to explore and play the games and the variety of games addressing the same math skills allow the children flexibility in their learning time and may contribute to their intellectual development by motivating them to build knowledge and constructively integrate thought, emotion and action [23], [62].

The virtual environment may contribute to a remediation of the different subtypes of number processing deficiencies and minimise the various symptoms of several deficits that constitute Dyscalculia (discussed in [8], [9], [11]). It presents the children with fun situational problems that teach mathematical skills relevant to children with learning disabilities in mathematics, especially children with Dyscalculia. Among these mathematical skills are the abilities to understand the concept of numbers and their size, to count, to sort and to perform arithmetic operations. In other words, the virtual environment exposes the child to fun situational problems that can be solved if the child develops a mathematical way of thinking. It also promotes the exchange of ideas, knowledge, plans and emotions between children through chat, contributing to their intellectual development [27], [28], [63].

The games developed specifically for Dyscalculia not only improved the specific mathematical skills that they were designed to address but also improved the other mathematical skills assessed by the SPT arithmetic sub-test.

Previous studies of the effects of games on mathematical skills showed some positive results, but these studies were limited.

The game “Number Race” [52], which worked on basic numerical cognition, was given to children with Dyscalculia between ages 7 and 9 for 30 minutes per day, on 4 days a week, until a maximum of 10 hours of training was reached (due to absences, the average was 8 hours of training) [43]. After the intervention and post-test, the authors concluded that the intervention improved the children's concept of numbers. However, that study did not have a control group. The effectiveness of the game “Graphogame-Math”, which works on exact numerosity and number symbols, was compared to the game “Number Race” when given to kindergarten children between 6 and 7 years old [39]. Daily 10–15 minute sessions were given for 3 weeks. The authors observed that the two groups of children described in the study had a significant improvement in number comparison skills but not in counting or arithmetic. Intervention with “Graphogame-Math” had a better result than “Number Race” for number comparison skills [39].

An adaptive E-learning tool that teaches children with Dyscalculia using an entertaining numerical comparison task [40] was also tested on nine children with mathematical learning disabilities, and the results showed that it may be useful for remediation of Dyscalculia in children age 8 and under. However, the study had several limitations: the lack of a control group; the small number of children with mathematical difficulties; and the lack of a detailed analysis of the children's learning improvements before and after the intervention.

The responses to the SPT post-test after the “Tom's Rescue” intervention showed that the children:

- Improved their capacity to say a sequence of numbers in increasing order, as all of the children in the experimental group correctly answered the item that tested number sequences on the SPT,
- Improved their arithmetic calculation procedures, as the EG children were able to solve simple arithmetic exercises,
- Improved their understanding of transcoding between verbal numbers and Arabic numerals.

## Conclusions

The purpose of this study was to test whether a fun virtual environment can improve math proficiency levels and motivate children with difficulties in math to confront challenges involving mathematical skills, which children with Dyscalculia normally have difficulties doing [7], [8], [21], [43], [55], [60], [61]. According to the literature, strategies embedded in entertaining games appear to motivate children more than those normally used in educational teaching games, suggesting that using entertaining game strategies could result in more efficient teaching [34], [41], [64].

The results showed that the objective of the virtual environment was achieved. Statistical analysis of the results showed that the virtual environment helped the EG significantly improve their scores on the post-test, whereas the CG did not improve their scores. The improvement in scores for children who had computer-based reinforcement compared with children who had reinforcement using traditional teaching confirmed that learning and training can change the human brain and generate measurable benefits for the children [24], [34], [65], [66]. The computer is an instigating, attractive and stimulating tool, in opposition to the use of notebook and blackboard of what children are used to. Thus, it is believed that using computer games in the teaching methods can stimulate the intellectual autonomy growth of these children [67], since some students that are difficult to motivate in the classroom or that have difficulty to perform certain tasks in traditional classes, surprisingly and actively participate in the teaching strategies that include ludic methods (plays and games) and/or the use of computer.

When the children were invited to play, they participated effectively in the game as a consequence from the children's immersion by the software [68], [69]. From that moment, the child establishes a relationship with the game that allows him to interact with the computer, through a graphical interface, that involves it using immediate feedbacks with visual and sound effects, challenges, storyline, plot and playfulness that only computerized games are able to offer [69], [26]. Thus, the child ceases to act as a passive subject starting to act actively. With the immersions, the child interacts with the reality of the game becoming represented by the figure of the avatar [68]. Thus, it is possible to observe that the software has the function of awaken the children's interest to interact with the character and to involve it to meet the challenges accepting the rules and limits proposed by the game [26].

The results were positive not only because the computer was enticing to the children, as observed by [23], [27], but also because of the learning strategy implemented in the virtual environment as suggest in [24]–[27], [34]. According to [70], children may stop playing when discouraged, which can occur when the implemented challenges are not exciting. The results showed that the fun nature of the virtual environment pleased all of the children, as a growing interest was recorded during the intervention sessions. In addition to playing the games more than once, they asked for the time to be extended so that they could continue playing. The teachers also commented that the children in the EG performed better in the classroom during and after the intervention. This is probably because interacting with the virtual environment led the children to be more willing to learn, as mentioned by [27], [26], and to develop necessary mathematical skills [24].The fact is that the ludic cannot be disregarded for exercising significant influence in the language, thinking and concentration development of the child [71], [72]. Through ludic the child has the curiosity stimulated [73], which leads to act and to acquire initiative to acting and self-confidence [74]. When playing, the child acts spontaneously and, when motived by challenge [75], the child uses all acquired knowledge besides creating new cognitive processes as she continues playing [76], [77], [78]. However, in the game, the act of making mistake is not negative, but just a stimulus to learning. During the game, the mistake can produce less frustrating results [79] and children can developed attention and concentration focus [80], [81].

The virtual environment was designed to incorporate distinct, entertaining games that address mathematical skills into a common storyline and to be played on the Internet with the possibility of interacting with other players through chat. The positive results of this virtual environment on mathematical skills show that pedagogical approaches implemented using games may help children learn and make efficient, flexible use of learning time if the games are connected to a virtual environment that permits free exploration without an established an order of execution. Therefore, using virtual environments similar to the ones shown in this study can help educators to create a teaching plan that improves their students' practical knowledge.
